# Non-*Saccharomyces* Yeasts Nitrogen Source Preferences: Impact on Sequential Fermentation and Wine Volatile Compounds Profile

**DOI:** 10.3389/fmicb.2017.02175

**Published:** 2017-11-06

**Authors:** Antoine Gobert, Raphaëlle Tourdot-Maréchal, Christophe Morge, Céline Sparrow, Youzhong Liu, Beatriz Quintanilla-Casas, Stefania Vichi, Hervé Alexandre

**Affiliations:** ^1^UMR Procédés Alimentaires et Microbiologiques, Université de Bourgogne Franche-Comté/AgroSup Dijon - Equipe VAlMiS (Vin, Aliment, Microbiologie, Stress), Institut Universitaire de la Vigne et du Vin Jules Guyot, Université de Bourgogne, Dijon, France; ^2^SAS Sofralab, Magenta, France; ^3^Nutrition, Food Science and Gastronomy Department, INSA - XaRTA (Catalonian Reference Network on Food Technology), University of Barcelona, Santa Coloma de Gramenet, Spain

**Keywords:** non-*Saccharomyces* yeasts, alcoholic fermentation, yeast interactions, nitrogen sources, volatile compounds, wine

## Abstract

Nitrogen sources in the must are important for yeast metabolism, growth, and performance, and wine volatile compounds profile. Yeast assimilable nitrogen (YAN) deficiencies in grape must are one of the main causes of stuck and sluggish fermentation. The nitrogen requirement of *Saccharomyces cerevisiae* metabolism has been described in detail. However, the YAN preferences of non-*Saccharomyces* yeasts remain unknown despite their increasingly widespread use in winemaking. Furthermore, the impact of nitrogen consumption by non-*Saccharomyces* yeasts on YAN availability, alcoholic performance and volatile compounds production by *S. cerevisiae* in sequential fermentation has been little studied. With a view to improving the use of non-*Saccharomyces* yeasts in winemaking, we studied the use of amino acids and ammonium by three strains of non-*Saccharomyces* yeasts (*Starmerella bacillaris, Metschnikowia pulcherrima*, and *Pichia membranifaciens*) in grape juice. We first determined which nitrogen sources were preferentially used by these yeasts in pure cultures at 28 and 20°C (because few data are available). We then carried out sequential fermentations at 20°C with *S. cerevisiae*, to assess the impact of the non-*Saccharomyces* yeasts on the availability of assimilable nitrogen for *S. cerevisiae*. Finally, 22 volatile compounds were quantified in sequential fermentation and their levels compared with those in pure cultures of *S. cerevisiae*. We report here, for the first time, that non-*Saccharomyces* yeasts have specific amino-acid consumption profiles. Histidine, methionine, threonine, and tyrosine were not consumed by *S. bacillaris*, aspartic acid was assimilated very slowly by *M. pulcherrima*, and glutamine was not assimilated by *P. membranifaciens*. By contrast, cysteine appeared to be a preferred nitrogen source for all non-*Saccharomyces* yeasts. In sequential fermentation, these specific profiles of amino-acid consumption by non-*Saccharomyces* yeasts may account for some of the interactions observed here, such as poorer performances of *S. cerevisiae* and volatile profile changes.

## Introduction

The main sources of yeast assimilable nitrogen (YAN) in grape must are ammonium and amino acids. YAN availability for winemaking is one of the major factors controlling alcoholic fermentation and preventing stuck or sluggish fermentations (Henschke and Jiranek, 1993; Alexandre and Charpentier, 1998).

Many studies have focused on the nitrogen requirements of the predominant yeast in the fermentation process*, Saccharomyces cerevisiae* (Beltran et al., 2002; Zott et al., 2008; Albergaria and Arneborg, 2016). Nitrogen demand is dependent on yeast strain, sugar content and fermentation conditions. It is generally agreed that at least 120–140 mg N/L YAN is required for satisfactory fermentation kinetics and final product quality (Bely et al., 1990; Jiranek et al., 1995). To prevent nitrogen deficiency, ammonium could be added which will influence biomass formation, fermentation kinetics, viability, and also the production of aroma compounds (Hernández-Orte et al., 2005, 2006; Hazelwood et al., 2008). The assimilation of diverse sources of nitrogen is tightly regulated. Nitrogen sources may be “good” (preferred nitrogen source) or poor (non-preferred nitrogen sources; Jiranek et al., 1995). Preferred nitrogen sources are assimilated more rapidly and are generally used earlier in the fermentation than poor nitrogen sources. These differences can be attributed principally to differences in the efficiency of the corresponding transport systems.

The pattern of nitrogen assimilation by *S. cerevisiae* in the presence of complex nitrogen sources is controlled by nitrogen catabolite repression (NCR). For example, ammonium is a preferred nitrogen source, and, when present in the medium, it represses the expression of catabolic pathways making use of other nitrogenous compounds (Ter Schure et al., 2000; Magasanik and Kaiser, 2002; Marks et al., 2003; Beltran et al., 2004). Permeases involved in the assimilation of preferred nitrogen sources are expressed whereas transporters of non-preferred nitrogen sources are repressed and degraded under NCR control (Ter Schure et al., 2000; Magasanik and Kaiser, 2002). At the start of the alcoholic fermentation, preferred nitrogen sources are present and NCR is active. As the concentrations of these preferred nitrogen sources decreases during fermentation, NCR weakens until a derepressed state is reached.

Many aroma compounds are directly related to nitrogen metabolism. For example, higher alcohols and their associated fatty acids and esters are influenced by the quality and quantity of nitrogen sources (Beltran et al., 2005; Hernández-Orte et al., 2005; Barbosa et al., 2009). Low nitrogen concentrations in grape directly affect higher alcohol production, with an inverse effect observed in the presence of moderate to high nitrogen levels (Äyräpää, 1968). Nitrogen limiting conditions lead to higher levels of higher alcohol production via both catabolic and anabolic biosynthetic pathways (Carrau et al., 2008). Furthermore, nitrogen metabolism also regulates other major pathways, such as sugar and sulfur metabolism, and the use of essential nutrients. It can affect the production of many flavor-active intermediates and end-products (Hernandez-Orte et al., 2006; Mendes-Ferreira et al., 2011; Hirst and Richter, 2016).

The use of non-*Saccharomyces* (NS) yeasts has spread in recent years, due to the value of these microbes for improving the aromatic profile of the wine (Lambrechts and Pretorius, 2000; Soden et al., 2000; Clemente-Jimenez et al., 2005; Sadoudi et al., 2012; Cordero-Bueso et al., 2013; Liu et al., 2016). The medium is generally either co-inoculated with these yeasts and *S. cerevisiae* (Anfang et al., 2009; Medina et al., 2013; Tofalo et al., 2016) or sequentially inoculated (Ciani et al., 2010; Liu et al., 2017; Puertas et al., 2017). However, very few studies have described YAN consumption by NS yeasts and its effects on the kinetics of fermentation by *S. cerevisiae*. Moreover, as many YAN sources are precursors of volatile molecules, YAN consumption by NS yeasts may affect the volatile compounds profile of the final product. In this study, we investigated the YAN consumption profiles of three NS yeast strains during alcoholic fermentation in different conditions. We then evaluated the effect of YAN consumption by these strains on the fermentation kinetics of *S. cerevisiae* in sequential fermentation conditions. Finally, the volatile compounds profiles of wines obtained in sequential fermentation were compared with the YAN consumption profiles.

## Materials and methods

### Microorganisms and media

Three non-*Saccharomyces* yeast strains isolated in Burgundy were obtained from the collection of the Burgundy University Vine and Wine Institute (*Institut Universitaire de la Vigne et du Vin*, IUVV). The strains selected for this study were *Starmerella bacillaris* (prev. *Candida zemplinina*) BBMV5FA17, *Metschnikowia pulcherrima* BB810, and *Pichia membranifaciens* BB3 (selection based on potential for use in the wine industry; Di Maio et al., 2012; Sadoudi et al., 2012; Cordero-Bueso et al., 2013; Liu et al., 2017). A commercial strain of *S. cerevisiae* “Selectys® La Marquise” (Sofralab, Magenta, France) was used as a reference and for sequential inoculation. Each strain was stored in 20% (v/v) glycerol at −80°C. Initial cultures for inoculation (precultures) were prepared by incubation in modified YPD medium (0.5% (w/v) yeast extract, 1% (w/v) peptone, 2% (w/v) glucose, and 0.02% (w/v) chloramphenicol) in 100 mL Erlenmeyer flasks at 28°C with stirring (150 rpm) for 24 h.

### Inoculation

The density of viable cells in precultures was determined by flow cytometry (BD Accuri™ C6). The fluorophore used to detect the viable cells was 5-6-carboxyfluorescein diacetate (cFDA) (Molecular Probes) dissolved in acetone at a final concentration of 500 μM. We added 10 μL of this solution to 100 μL of diluted yeast suspension. Before measurement, the suspension was incubated in the dark for 20 min. Once the density of viable cells had been determined, we sampled 10^6^ or 5 × 10^6^ viable cells/mL. This sample was centrifuged at 10 000 × g. The supernatant was discarded and the pellet was resuspended in grape juice and used to inoculate 200 mL of grape juice for the fermentation.

### Fermentation conditions

All fermentations were carried out with the same batch of commercial grape juice (100% pure white and Muscat grape juice from the industry with initial concentration of 150 g/L of sugar) supplemented with equal proportions of glucose and fructose to achieve a final sugar concentration of 200 g/L. This grape juice was sterilized by filtration (0.22 μm pores). We used 250 mL Erlenmeyer flasks containing 200 mL of sterile grape juice covered with sterile cotton wool to prevent contamination. For pure cultures, fermentations were performed under three sets of conditions: at 28°C with shaking (150 rpm); at 28°C without shaking; at 20°C without shaking. For sequential fermentations, cultures were incubated at 20°C without shaking. Samples were collected at 6-h intervals during the first 3 days, and then every 24 h for fermentations performed at 28°C and after 24 h only for fermentations at 20°C. For pure cultures, the medium was inoculated with 10^6^ viable cells/mL of NS yeast or *S. cerevisiae*. For sequential inoculations, the medium was inoculated with 5 × 10^6^ viable cells of NS yeast/mL. After 3 days of fermentation, the medium was inoculated with 10^6^ viable cells of *S. cerevisiae*/mL. Cultures at 28°C were conducted in duplicate, and cultures at 20°C (pure and sequential) were conducted in triplicate.

### Monitoring of biomass growth

For pure cultures, viable cell density was determined as before inoculation. For sequential fermentations, serial dilutions of cell suspensions in 0.9% (w/v) NaCl were generated. Samples of each dilution (100 μL) were then spread on solid lysine medium (Oxoid LTD., England) and incubated at 28°C for 3 or 4 days. On this medium, NS yeast colonies appear rapidly and are clearly visible, whereas *S. cerevisiae* “Selectys® La Marquise” (SLM) colonies appear more slowly and are smaller. Unlike flow cytometry, here only cultivable cells are detected.

### Analytical methods

#### Enological analysis

Samples were centrifuged at 10,000 × g for 5 min and the supernatants were stored at −20°C until analysis. Total sugar and ethanol concentrations were determined by FTIR spectroscopy with an OenoFoss type 4101 apparatus (FOSS Electric, Denmark).

#### Concentrations of amino acids and ammonium

Amino acids and ammonium were derivatized with an AccQ-Tag™ Ultra Derivatization kit, according to the manufacturer's instructions (Waters, USA). Separation was then performed by high-performance liquid chromatography (HPLC) with a C18 reverse-phase column (AccQ-Tag™ Ultra Column, 3.9 × 150 mm) and fluorometric detection. L-alpha-amino-*n*-butyric acid (0.1 mM) was added to the samples, as an internal standard, as previously described (Ritt et al., 2009). For each sample, we injected 1 μL onto the column and the chamber was maintained at 37°C. The buffer flow rate was 1 mL/min. The sample was eluted according to the gradient program shown in Table 1. Buffer A contained 140 mM sodium acetate trihydrate, 6.9 mM triethylamine, and 3.42 μM ethylenediaminetetraacetic acid. Buffer B contained 99.8% acetonitrile. Both buffers were filtered (0.22 μm pores) before use.

**Table 1:** Gradient program for HPLC separation and the quantification of amino acids and ammonium.

**Time (min)**	**Buffer A (%)**	**Buffer B (%)**	**Water (%)**
0	100	0	0
1	100	0	0
1.5	99	1	0
16.5	97	3	0
25.5	94	6	0
35.5	86	14	0
51.5	86	14	0
52	0	60	40
55	0	60	40
55.5	100	0	0
66.5	100	0	0

#### HS-SPME-GC/MS analysis of volatile compounds

We placed 2 mL of wine in a 10 mL vial fitted with a silicone septum, which was then transferred to a silicon oil bath at 40°C, in which the sample was incubated for 10 min, with magnetic stirring (700 rpmA divinylbenzene/carboxen/polydimethylsiloxane (DVB/CAR/PDMS) fiber (Supelco, Bellefonte, PA, USA) was exposed to the sample headspace for 30 min and then subjected to immediate desorption in the gas chromatograph injector.

Volatile compounds were analyzed by gas chromatography coupled to quadrupolar mass selective spectrometry with an Agilent 5973 Network detector (Agilent Technologies, Palo Alto, CA, USA). Analytes were separated on a Supelcowax-10 (Supelco) column with a length of 60 m, an internal diameter of 0.25 mm and a film thickness of 0.25 μm. Column temperature was held at 40°C for 5 min, increased to 130°C at a rate of 4.5°C/min, and then to 250°C at 15°C/min. The temperature was then held at 250°C for 2 min. The injector temperature was 260°C and the time fiber desorption time in the injection port was fixed at 5 min. Helium at a flow rate of 1.2 mL/min was used as the carrier gas. Ion source temperature was 175°C and transfer line temperature was 280°C. Electron impact mass spectra were recorded at an ionization energy of 70 eV, with 2 scan/s. GC–MS analysis was performed in complete scanning mode (SCAN), in the 30–300 mass units range.

Compounds were identified by comparison of their mass spectra and retention times with those of standard compounds or with those available in the Wiley 6 mass spectrum library or reported in previous publications. Response factors were calculated for volatile compounds from calibration curves obtained by analyzing hydroalcoholic solutions (ethanol 10%, v/v) of various reference compounds in the 2–500 μg/L (ethyl hexanoate, ethyl octanoate, ethyl decanoate, 1-hexanol, isoamylacetate, and linalyl oxide) and 1–50 mg/L (3-methylbutanol, phenylethyl alcohol, and octanoic acid) concentration ranges.

### Comparison of the kinetics of nitrogen consumption

T_50_ values were used for the comparison of YAN consumption rates. This value is the time point at which 50% of the initial concentration of the YAN is consumed. It was determined by fitting a logistic function (Equation 1) to nitrogen consumption curves with OriginPro8 version 8E.

**Equation 1:** Fitting equation for the determination of T_50_. A1: Maximum amino acid or ammonium concentration, A2: Residual amino acid or ammonium concentration, x: time, x0: T_50_, P: coefficient.

$$\begin{array}{l} y=\frac{A1-A2}{1+\left(\frac{x}{x0}\right)p}+A_{2} \end{array}$$

### Statistical analysis

Preferred amino acid groups were compared in the Kruskal–Wallis test, an alternative to ANOVA, with XLSTAT version 2014 statistical software (Addinsoft, France). Differences in aroma compound concentrations were assessed by analysis of variance (Tukey's test). Differences were considered significant if the *P-*values obtained were below 0.05. For the analysis of multivariate data principal component analysis (PCA) was constructed performed with XLSTAT version 2014 statistical software (Addinsoft, France). For the analysis of other data, we also used Microsoft Excel 2013 and OriginPro8 version 8E.

## Results

### Profile of the consumption of nitrogen sources by NS yeasts

**Figure 1:**
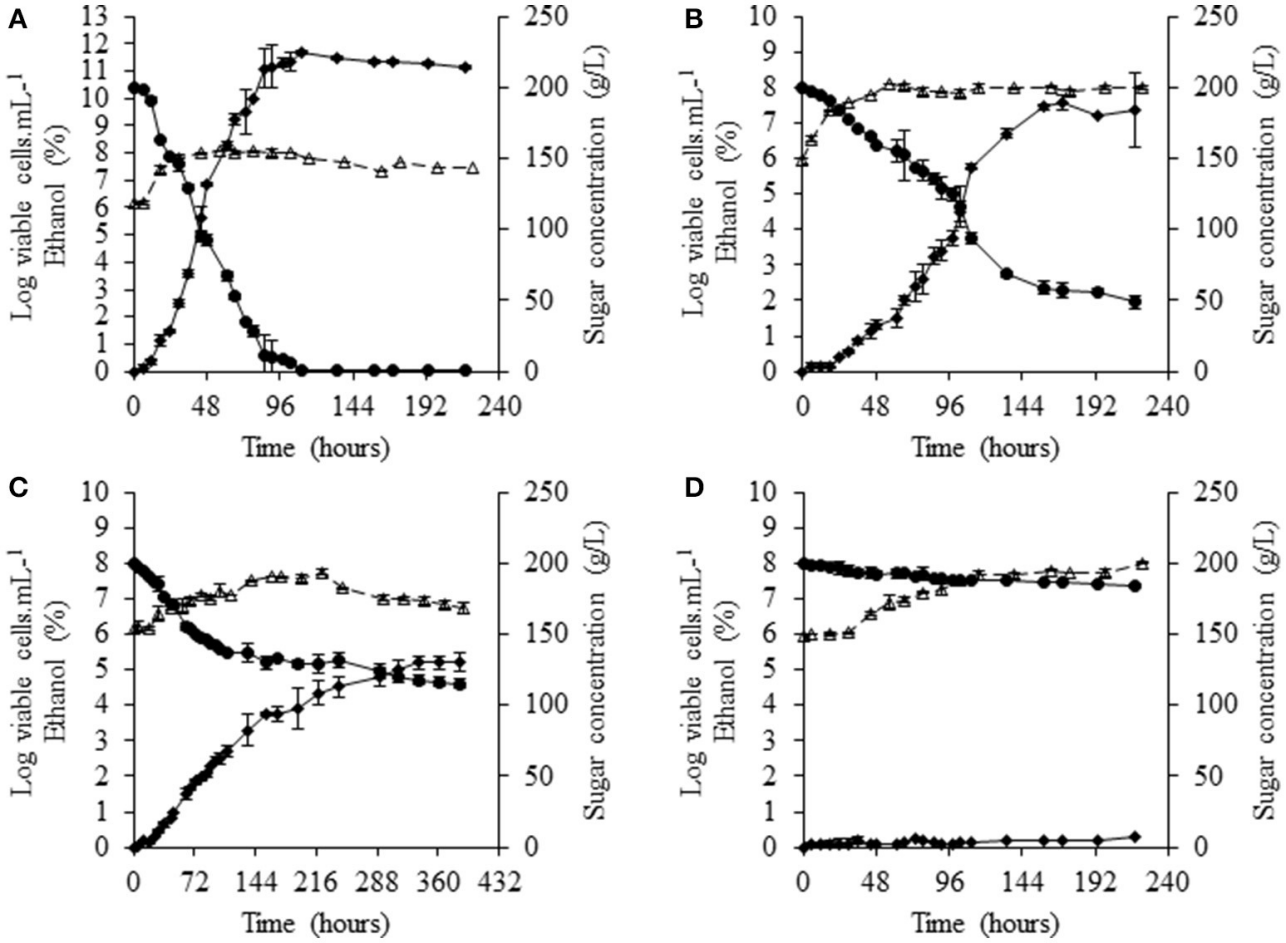
Fermentation profiles of selected NS yeasts in grape juice at 28°C. **(A)** *S. cerevisiae* SLM, **(B)** *S. bacillaris* BBMV5FA17, **(C)** *M. pulcherrima* BB810, and **(D)** *P. membranifaciens* BB3. Dotted curves with white triangles represent viable populations, solid curves with black circles show sugar concentration and solid curves with black diamonds indicate the concentration of ethanol. For each strain, the experiments were performed in duplicate and the error bars represent the standard deviation of the results.

Monocultures were performed in commercial grape juice containing 200 g/L sugar and 450 mg/L YAN. At 28°C, the reference *S. cerevisiae* strain, SLM, completed fermentation in 134 h, consuming all the sugar present, resulting in the formation of about 10^8^ cells/mL (Figure 1A). Fermentations with NS yeasts were slower. In 150 h, a large amount of the sugar present (150 g/L) was consumed by *S. bacillaris* BBMV5FA17 but, thereafter, sugar consumption was significantly lower (Figure 1B). Biomass formation was similar to that with *S. cerevisiae* SLM. *M. pulcherrima* BB810 consumed significantly less sugar than *S. bacillaris* BBMV5FA17, with only 70 g/L consumed in 158 h. The biomass generated reached ~3.1 × 10^7^ cells/mL (Figure 1C). *P. membranifaciens* BB3 was not a good fermenting yeast, as it consumed only 16 g/L sugar over the course of the experiment and formed 6.3 × 10^7^ cells/mL (Figure 1D). At 20°C, the total NS yeast population varied from 1.9 × 10^7^ cells/mL for *M. pulcherrima* BB810 (Supplementary Figure 1C) to 2.5 × 10^7^ cells/mL for *P. membranifaciens* BB3 (Supplementary Figure 1D), whereas that for *S. bacillaris* BBMV5FA17 (Supplementary Figure 1B) reached ~10^8^ cells/mL like *S. cerevisiae* SLM (Supplementary Figure 1A). None of the NS strains performed a complete fermentation in any of the conditions tested (28 or 20°C), over the duration of the experiment.

**Figure 2:**
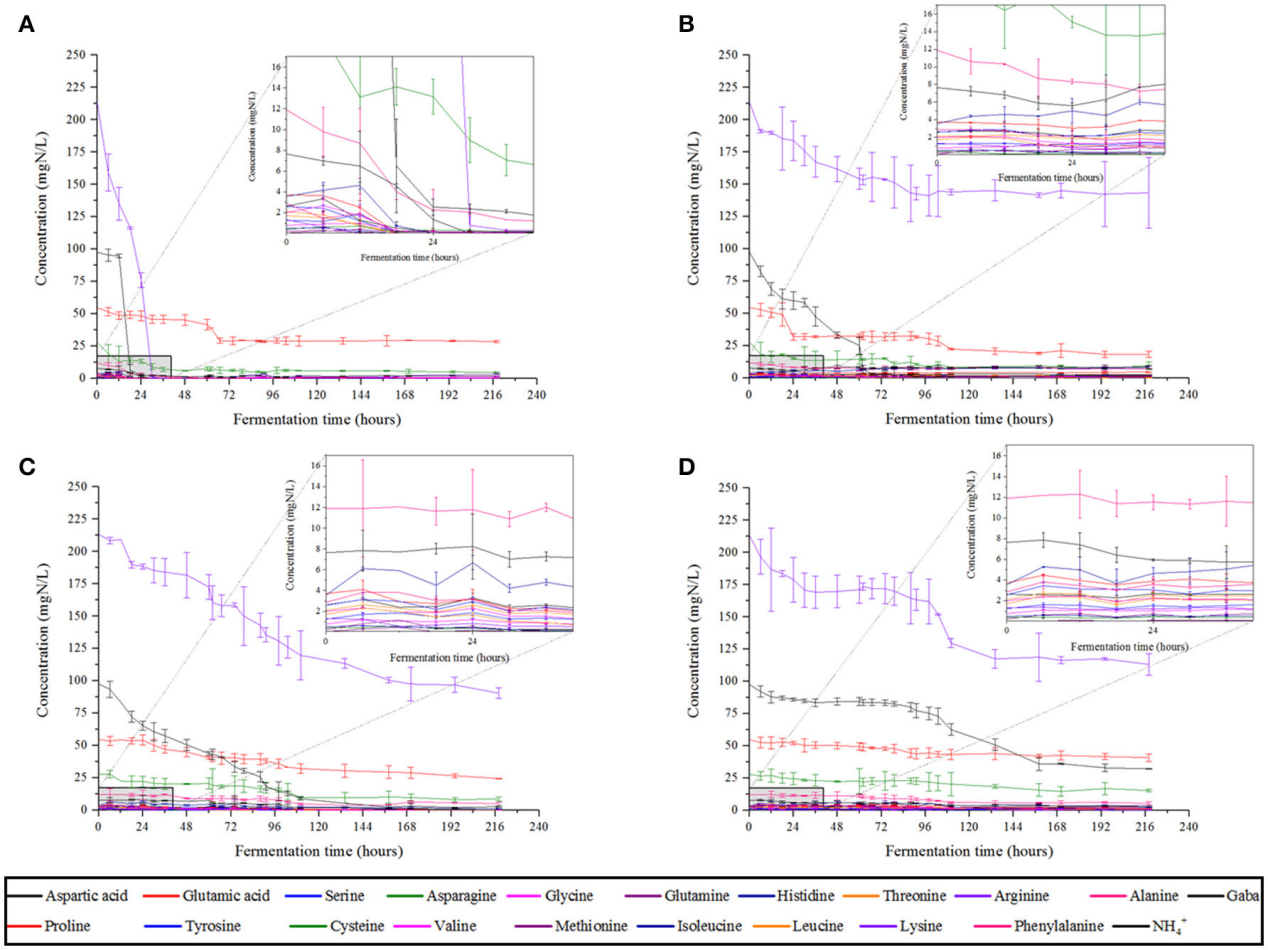
YAN consumption profiles of *S. cerevisiae* SLM **(A)**, *S. bacillaris* BBMV5FA17 **(B)**, *M. pulcherrima* BB810 **(C)**, and *P. membranifaciens* BB3 **(D)** in grape juice at 28°C. For each strain, the experiments were performed in duplicate and the error bars represent the standard deviation of the results.

We monitored YAN consumption at both culture temperatures. At 28°C, most of the YAN was consumed within 30 h by *S. cerevisiae* SLM, but only 10 mg/L proline was consumed in 72 h (Figure 2A). Consumption occurred during the exponential growth phase of the yeast (Figure 1A). NS yeasts generally consumed YAN more slowly than *S. cerevisiae. S. cerevisiae* SLM consumed almost all the YAN present (396 mg/L) in 72 h, whereas *S. bacillaris* BBMV5FA17 consumed 193 mg/L, *M. pulcherrima* BB810 168 mg/L and *P. membranifaciens* BB3 70 mg/L. These observations may reflect the lower biomass production and lower growth rate of NS yeasts, because *S. bacillaris* BBMV5FA17 took 45 h to reach exponential growth phase, *M. pulcherrima* BB810 took 57 h and *P. membranifaciens* BB3 took 115 h. In addition to these differences in kinetics, the nature of the YAN consumed differed between NS yeasts and *S. cerevisiae* SLM. The predominant YAN sources (arginine, ammonium, and asparagine) were consumed within 30 h by *S. cerevisiae* SLM, whereas *S. bacillaris* BBMV5FA17 consumed arginine more slowly, in accordance with its slower growth rate (70 mg/L in 218 h; Figure 2B). *M. pulcherrima* BB810 (Figure 2C) and *P. membrnifaciens* BB3 (Figure 2D) also consumed this amino acid more slowly than *S. cerevisiae* SLM. Ammonium was rapidly completely consumed by *S. bacillaris* BBMV5FA17 (97 mg/L in 72 h), but more slowly by *M. pulcherrima* BB810 (complete consumption in 158 h), and it had not been completely consumed by *P. membranifaciens* BB3 by the end of the experiment. The overall YAN consumption rate was lower at 20°C, for all strains, with some variation of preferential nitrogen sources (Supplementary Figure 2).

We therefore used T_50_ values to identify the sources preferred by the different yeasts. In our classification, group A corresponds to preferred sources, B to intermediate sources, C to non-preferred sources and D or ^*^ indicates very low levels of assimilation or no assimilation, respectively. This classification was applied to fermentations performed at 28°C (Supplementary Figure 3) and at 20°C (Supplementary Figure 4). A summary of the YAN sources used more by NS yeasts than by *S. cerevisiae* SLM at the two temperatures is provided in Table 2. Strikingly, histidine, methionine, threonine and tyrosine were not consumed by *S. bacillaris* BBMV5FA17, at either temperature. Aspartic acid was assimilated very slowly (group D) or was a non-preferred source (group C) for *M. pulcherrima* BB810, whereas this amino acid was well-assimilated by *S. cerevisiae* SLM (group A or B). Glutamine was not assimilated by *P. membranifaciens* BB3. By contrast, cysteine appeared to be a preferred source of YAN for all NS yeasts whereas it was assigned to group C for *S. cerevisiae* SLM.

**Table 2:** Overview of YAN ranking on the basis of consumption rate.

**YAN**	**28°C**				**20°C**			
	**Sc**	**Sb**	**Mp**	**Pm**	**Sc**	**Sb**	**Mp**	**Pm**
Aspartic acid	A	^*^	D	C	B	B	C	^*^
Glutamic acid	B	^*^	C	C	B	B	B	B
Alanine	B	A	C	B	C	A	A	B
Arginine	C	B	D	B	C	B	D	C
Asparagine	A	D	B	A	A	B	^*^	^*^
Cysteine	C	A	A	A	C	A	A	A
GABA	C	^*^	C	B	A	A	C	B
Glutamine	A	^*^	A	^*^	B	B	A	^*^
Glycine	C	^*^	D	^*^	A	A	B	A
Histidine	C	^*^	C	^*^	A	^*^	A	A
Isoleucine	A	B	A	C	B	B	B	B
Leucine	A	B	A	B	C	B	B	C
Lysine	A	^*^	A	B	A	A	A	A
Methionine	B	^*^	A	^*^	A	^*^	^*^	^*^
${\text{NH}}_{4}^{+}$	B	B	B	C	B	B	C	D
Phenylalanine	B	B	B	C	A	B	C	B
Proline	C	C	C	A	C	C	^*^	^*^
Serine	A	B	C	A	C	B	C	^*^
Threonine	A	^*^	C	^*^	A	^*^	A	A
Tyrosine	C	^*^	D	C	B	^*^	D	B
Valine	B	B	B	C	C	C	C	^*^

Preferred sources are indicated by A, intermediate sources by B, non-preferred sources by C, very low levels of assimilation by D and an absence of assimilation by ^*^. Classification was based on the T_50_ values obtained. The different yeasts are represented as Sc for S. cerevisiae SLM, Sb for S. bacillaris BBMV5FA17, Mp for M. pulcherrima BB810 and Pm for P. membranifaciens BB3.

Temperature had a marked effect. *S. bacillaris* BBMV5FA17 did not consume GABA, glycine, and lysine at 28°C, whereas these molecules were preferred sources (group A) at 20°C. The same pattern was observed in *M. pulcherrima* BB810 for alanine and histidine (group C at 28°C and group A at 20°C) and in *P. membranifaciens* BB3 for glycine, histidine and threonine (non-assimilated at 28°C and group A at 20°C). With the exception of consumption or non-consumption, the classification of an individual YAN could change from preferred source (A) or intermediate group (B) to non-preferred source (C), and vice versa, in different conditions. These changes highlight the complexity of the classification.

These results show, for the first time, that the NS strains studied here can consume some of the YAN, thereby modifying the quantity and quality of the nitrogen sources present in the grape juice. These results are of particular importance for spontaneous and sequential fermentations. Indeed, NS yeasts develop first and may therefore consume nitrogen compounds that may affect fermentation kinetics and the aroma profile of the wine. We performed sequential fermentations to investigate the impact of YAN consumption by NS yeasts.

### Consequence of sequential fermentations with NS yeasts on YAN sources and volatile compounds profiles

**Figure 3:**
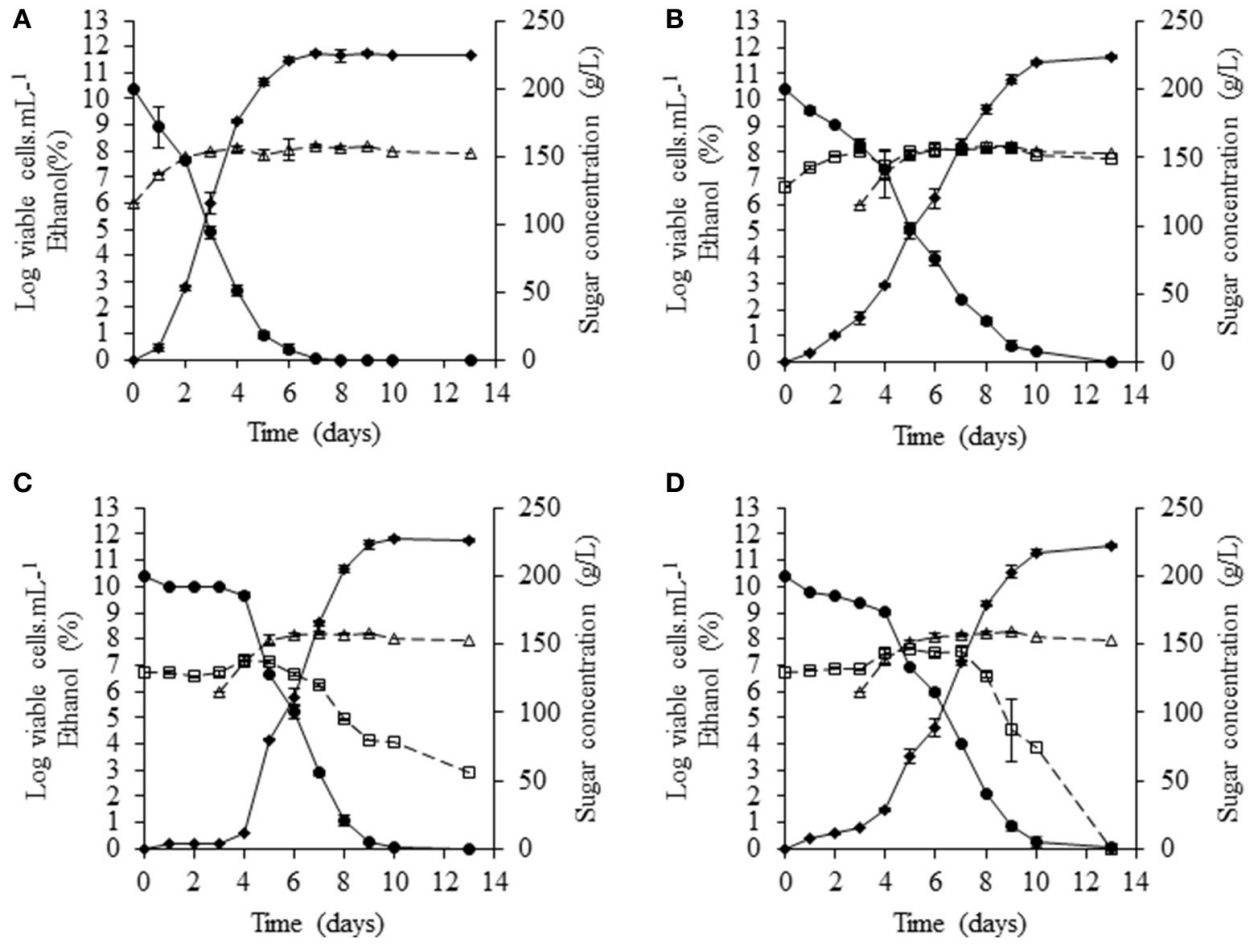
Sequential fermentation profiles of selected NS yeasts (white squares) with *S. cerevisiae* (white triangles) in grape juice in static conditions at 20°C.**(A)** *S. cerevisiae* SLM (control), **(B)** *S. bacillaris* BBMV5FA17—*S. cerevisiae* SLM, **(C)** *M. pulcherrima* BB810—*S. cerevisiae* SLM and **(D)** *P. membranifaciens* BB3—*S. cerevisiae* SLM. Dotted curves represent cultivable populations, solid curves with black circles show sugar concentration and solid curves with black diamonds indicate the concentration of ethanol. For each strain, the experiments were performed in triplicate and the error bars represent the standard deviation of the results.

Fermentations were carried out at 20°C, with the same grape juice used for pure cultures. NS yeasts were inoculated at a density of 5 × 10^6^ cells/mL and, after 3 days of fermentation, *S. cerevisiae* SLM was added at a density of 10^6^ cells/mL. *S. cerevisiae* SLM in pure culture performed complete fermentation in 7 days, consuming all the sugar present and generating a biomass of 10^8^ cells/mL (Figure 3A). In sequential fermentations, only *S. bacillaris* BBMV5FA17 remained cultivable throughout the fermentation (Figure 3B), generating amounts of biomass similar to those produced by *S. cerevisiae* SLM (~10^8^ cells/mL). In these conditions, sugar consumption increased slightly 1 day after inoculation of the medium with *S. cerevisiae*. This effect was even more marked in sequential fermentations with *M. pulcherrima* BB810 and *P. membranifaciens* BB3 (Figures 3C,D, respectively). Furthermore, the viability of *M. pulcherrima* BB810 and *P. membranifaciens* BB3 decreased significantly (Figures 3C,D) 2 days after the addition of *S. cerevisiae*. Cell cultivability decreased particularly rapidly for *P. membranifaciens* BB3, for which no cultivable cells were present 9 days after the addition of *S. cerevisiae* SLM.

**Figure 4:**
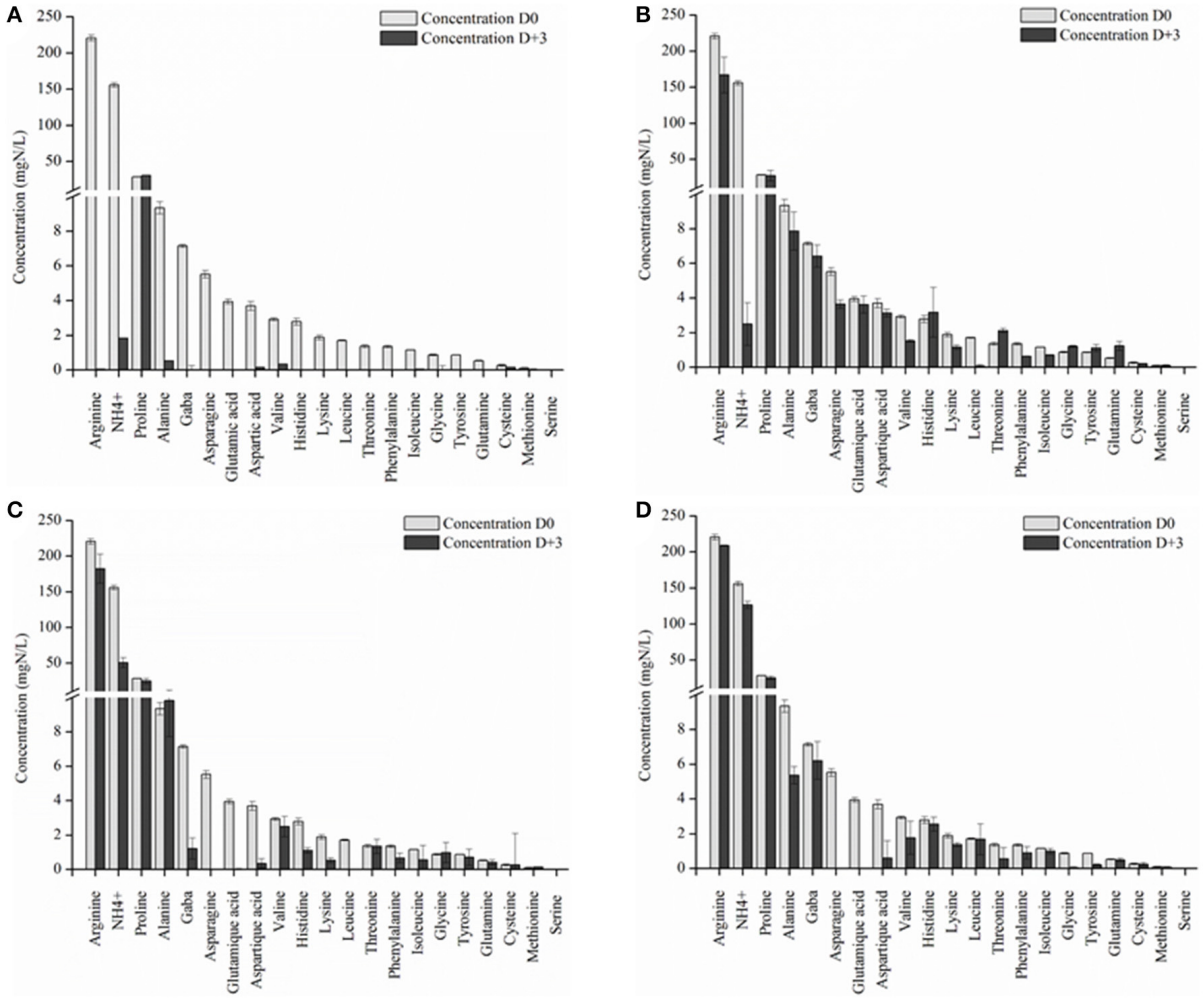
YAN concentration in grape juice initially (D0) and after 3 days of fermentation (D+3) by *S. cerevisiae* SLM **(A)**, *S. bacillaris* BBMV5FA17 **(B)**, *M. pulcherrima* BB810 **(C)**, and *P. membranifaciens* **(D)**. For each strain, the experiments were performed in triplicate and the error bars represent the standard deviation of the results.

**Figure 5:**
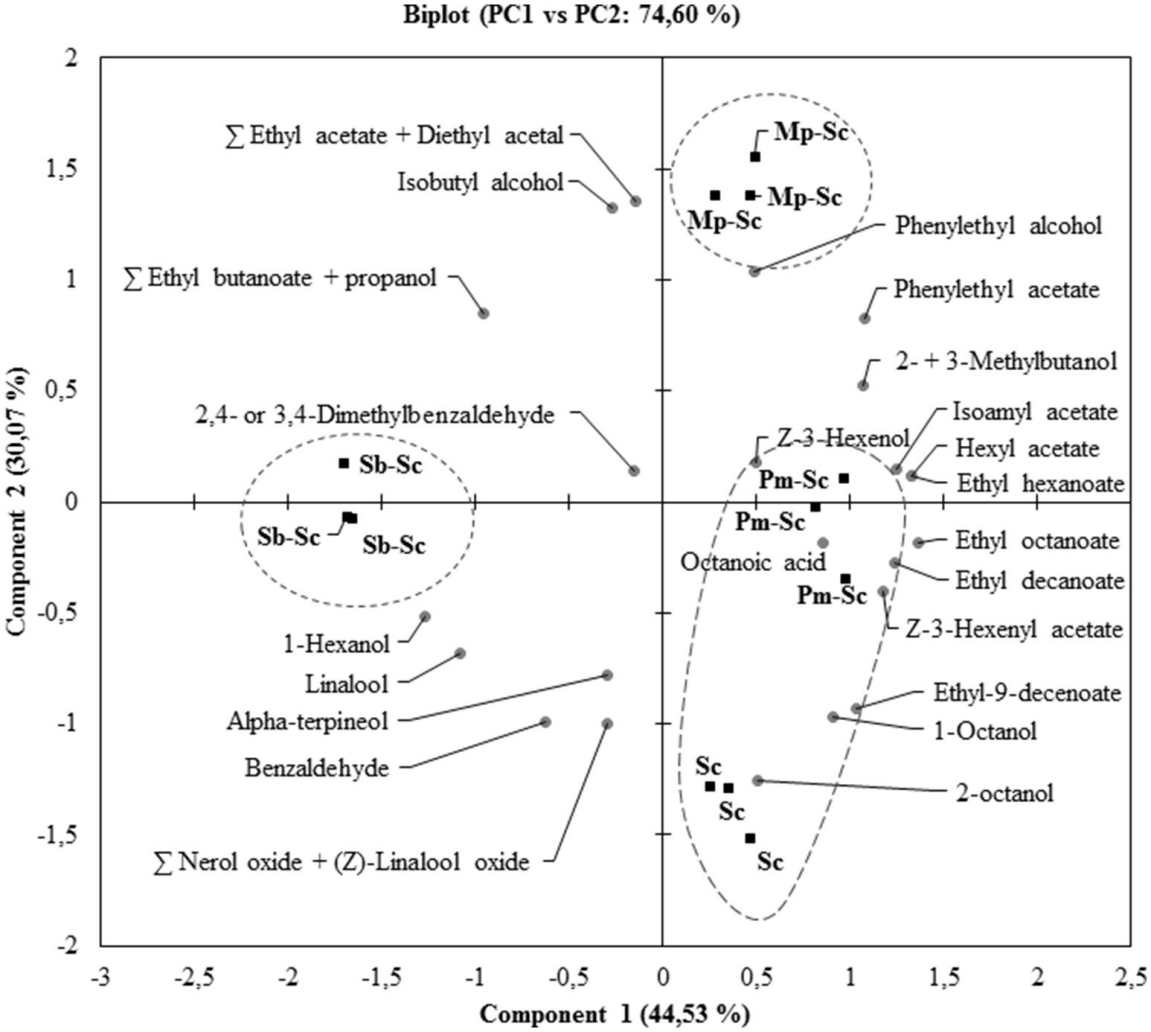
Biplot of the principal component analysis (PC1 vs. PC2) for volatile compounds **(A)** from sequential fermentations: *S. bacillaris* BBMV5FA17—*S. cerevisiae* SLM **(Sb–Sc)**, *M. pulcherrima* BB810—*S. cerevisiae* SLM **(Mp–Sc)**, *P. membranifaciens* BB3—*S. cerevisiae* SLM **(Pm–Sc)** at 20°C. *S. cerevisiae* SLM **(Sc)** in pure culture was used as a control. Ellipses represent clusters obtained from HCA.

The concentration of YAN available for *S. cerevisiae* SLM at the time of inoculation depended on the NS yeast strains. First, in three days, *S. cerevisiae* SLM consumed all YAN excepted proline as expected (Figure 4A). *S. bacillaris* BBMV5FA17 consumed 215 mg/L of the 450 mg/L YAN initially present, whereas *M. pulcherrima* BB810 and *P. membranifaciens* BB3 consumed 173 mg/L and 66 mg/L YAN, respectively. YAN matrix composition and quantity were therefore different between cultures inoculated with *S. cerevisiae* alone and those inoculated with *S. cerevisiae* 3 days after NS yeasts. During the first step in the sequential fermentation, large amounts of ammonium and leucine were consumed by *S. bacillaris* BBMV5FA17 (Figure 4B). *M. pulcherrima* BB810 consumed mostly GABA, asparagine, glutamic acid, aspartic acid, and leucine (Figure 4C). For *P. membranifaciens* BB3, the YAN sources consumed were asparagine, glutamic acid, aspartic acid, and glycine (Figure 4D). YAN sources have been identified as potential volatile compounds precursors in *S. cerevisiae* (Hazelwood et al., 2008) and the NS yeasts used in sequential fermentation clearly influence the volatile compounds profile of the wine produced. We compared the YAN profiles obtained in sequential fermentations with the volatile compounds profile of the wine, by HS-SPME-GC/MS analysis for each sample. We identified 22 volatile compounds in total (Table 3). The major changes relative to *S. cerevisiae* SLM concerned the sum of the concentrations of ethyl acetate + diethyl acetal and 1-octanol. The concentrations of these volatile compounds were significantly lower in sequential fermentations with all NS yeasts. However, isobutyl alcohol and phenylethyl acetate concentrations were higher in all sequential fermentations. We performed principal component analysis (PCA) to gain further insight (Figure 5). For the first two principal components accounted for 74.60% of the total variance s. A classical ascendant hierarchy method identified three groups on the basis of significant differences in aroma production. Several molecules derived from yeast metabolism were present at significantly lower concentrations in sequential fermentation with *S. bacillaris* BBMV5FA17 than in pure cultures of *S. cerevisiae* SLM. These decreases could may result from lower YAN availability to *S. cerevisiae* SLM. The lower isoamyl acetate concentration in sequential fermentations involving *S. bacillaris* BBMV5FA17/*S. cerevisiae* SLM may be directly linked to the consumption of leucine by *S. bacillaris* BBMV5FA17 (which produces small amounts of this molecule) during the first stage of fermentation. Leucine was also totally consumed by *M. pulcherrima* BB810 (Figure 4C), but in this case, isoamyl acetate concentration was similar to that in pure cultures of *S. cerevisiae* SLM (Table 3). Conversely, leucine was not consumed by *P. membranifaciens* BB3 during the 3 days of fermentation (Figure 4D). Isoamyl acetate concentration in sequential cultures with this NS yeast was very similar to that in pure cultures of *S. cerevisiae* SLM (Table 3). Therefore, in this case, the isoamyl acetate present was probably mostly produced by *S. cerevisiae* SLM.

**Table 3:** Concentration of volatile constituents in the wine (μg/L) after sequential fermentations of *S. bacillaris* BBMV5FA17—*S. cerevisiae* SLM (Sb–Sc), *M. pulcherrima* BB810—*S. cerevisiae* SLM (Mp–Sc), *P. membranifaciens* BB3—*S. cerevisiae* SLM (Pm–Sc) at 20°C in static conditions.

**Compounds**	**Sc**	**Sb-Sc**	**Mp-Sc**	**Pm-Sc**
∑ Ethyl acetate + diethyl acetal^*^	434.2 ± 61.4^a^	1051.5 ± 38.2^b^	1605.4 ± 140.0^c^	837.4 ± 39.6^d^
∑ Ethyl butanoate + propanol^*^	92.2 ± 40.7^c^	223.4 ± 15.5^a^	182.6 ± 6.9a.^b^	136.1 ± 24.6^b.c^
Isobutyl alcohol^*^	1102.9 ± 292.6^c^	2088.0 ± 225.2^b^	2740.7 ± 188.2^a^	1649.4 ± 123.5^b.c^
Isoamyl acetate^*^	468.2 ± 194.1^a^	21.8 ± 3.4^b^	561.9 ± 80.2^a^	509.8 ± 146.5^a^
2- + 3-Methylbutanol^*^	24515.8 ± 3420.4^a.b^	16541.0 ± 608.3^c^	29735.3 ± 1568.3^a^	24155.0 ± 1563.3^b^
Ethyl hexanoate^**^	72.8 ± 8.9^a^	29.2 ± 3.9^b^	78.5 ± 11.8^a^	81.9 ± 7.1^a^
Hexyl acetate^**^	38.7 ± 4.0^a^	18.9 ± 1.7^b^	41.2 ± 5.3^a^	42.8 ± 3.2^a^
Z-3-Hexenyl acetate^*^	4.7 ± 1.0^b^	nd	2.7 ± 0.5^b^	8.0 ± 1.6^a^
1-Hexanol^**^	203.0 ± 20.9^b^	276.5 ± 8.7^a^	143.5 ± 2.3^c^	149.9 ± 6.8^c^
Z-3-Hexenol^*^	32.3 ± 8.2^a^	25.2 ± 3.1^a^	34.2 ± 5.8^a^	29.5 ± 7.4^a^
Ethyl octanoate^*^	73.9 ± 8.2^b^	7.9 ± 1.7^c^	64.4 ± 8.5^b^	94.6 ± 7.5^a^
∑ Nerol oxide + (Z)-Linalool oxide^*^	27.1 ± 3.1^a^	23.3 ± 1.1^a.b^	19.7 ± 1.0^b^	19.8 ± 3.9^b^
2-octanol^*^	41.6 ± 3.4^a^	22.0 ± 3.6^b^	16.4 ± 0.7^b^	33.1 ± 4.2^a^
Linalool^*^	18.0 ± 1.1^a.b^	21.0 ± 1.4^a^	13.8 ± 0.8^c^	14.6 ± 2.2^b.c^
Benzaldehyde^*^	9.5 ± 2.7^a^	7.6 ± 0.7^a^	2.8 ± 0.3^b^	2.5 ± 0.7^b^
1-Octanol^*^	59.2 ± 3.2^a^	12.3 ± 1.8^b^	22.7 ± 2.4^c^	41.2 ± 4.9^d^
Ethyl decanoate^*^	12.9 ± 1.3^a^	7.1 ± 0.3^b^	11.7 ± 1.8^a^	12.8 ± 1.1^a^
Ethyl-9-decenoate^***^	17.2 ± 1.1^a^	3.4 ± 0.3^c^	6.6 ± 1.4^b^	15.1 ± 0.7^a^
Phenylethyl acetate^**^	9.0 ± 0.9^c^	2.4 ± 0.1^d^	20.9 ± 1.2^a^	14.6 ± 2.0^b^
2.4- or 3.4-Dimethylbenzaldehyde^*^	122.0 ± 5.3^a^	117.7 ± 3.8^a^	126.3 ± 10.2^a^	101.0 ± 37.9^a^
Phenylethyl alcohol^***^	19205.9 ± 1668.9^b^	14003.5 ± 1193.8^b^	45648.6 ± 2397.7^a^	17394.9 ± 2704.5^b^
Octanoic acid^***^	179.4 ± 14.8^b^	120.3 ± 13.9^b^	140.1 ± 11.9^b^	438.2 ± 75.6^a^

*Values with the same letters were not significantly different in Tukey's test (95%)*.

^***^Significance level for one-way ANOVA (

* 0.05 > p > 0.01;

** 0.01 > p > 0.001;

*** *p < 0.001)*,

*nd, not detected. S. cerevisiae SLM (Sc) in pure culture was used as a control. The data shown are the means of three replicates ± standard deviations*.

In some cases, sequential fermentation led to an increase in the concentration of some volatile compounds. Indeed, sequential fermentation with *S. bacillaris* BBMV5FA17 and *S. cerevisiae* SLM led to an increase in the production of ethyl butanoate + propanol, isobutyl alcohol and 1-hexanol. Sequential fermentation with *M. pulcherrima* BB810 and *S. cerevisiae* SLM significantly increased the concentrations of ethyl butanoate + propanol, isobutyl alcohol (more than *S. bacillaris* BBMV5FA17), phenylethyl alcohol and phenylethyl acetate. Finally, with *P. membranifaciens* BB3, the differences between pure cultures of *S. cerevisiae* SLM and sequential fermentation were observed essentially for Z-3-hexenyl acetate, ethyl octanoate, phenylethyl acetate, and octanoic acid (Table 3). Despite a probable synergistic effect on the production of some volatile compounds in sequential fermentations of *P. membranifaciens* BB3 with *S. cerevisiae* SLM, PCA analysis was unable to distinguish between the volatile compounds profiles of *S. cerevisiae* SLM alone and the sequential fermentation. The volatile compounds profiles of *S. cerevisiae* SLM and the *P. membranifaciens* BB3/*S. cerevisiae* SLM sequential fermentation were found in the same cluster as the other sequential fermentations.

## Discussion

NS yeasts now have several industrial applications. Their use in sequential fermentations and cofermentations is recommended in wine-making, to improve the aromatic profile of the wine (Romano et al., 2003; Anfang et al., 2009; Andorrà et al., 2010; Sadoudi et al., 2012; Jolly et al., 2014; Liu et al., 2016; Mas et al., 2016; Tofalo et al., 2016; Varela, 2016; Puertas et al., 2017; Whitener et al., 2017). However, it remains unclear how NS yeasts effect these positive changes. NS yeasts predominate during the early stages of fermentation (Zott et al., 2008; Liu et al., 2015), but their populations gradually decrease or disappear, to be replaced by *S. cerevisiae*, the yeast with the highest fermentation capacity (Wang et al., 2016). At the start of fermentation, NS yeasts consume some of the nutrients present, including YAN, which is known to be a source of volatile compounds precursors in *S. cerevisiae* (Hazelwood et al., 2008). Several studies have shown that *S. cerevisiae* has preferences among nitrogen sources (Jiranek et al., 1995; Crépin et al., 2012), but little is known about the nitrogen source preferences of NS yeasts (Andorrà et al., 2010; Kemsawasd et al., 2015). We addressed this issue, with a view to improving the management of NS yeast implementation, by determining the YAN consumption profiles of NS yeasts in pure culture and quantifying aroma compound production in sequential fermentations with *S. cerevisiae*. YAN sources were classified into groups according to the preferences of the three NS yeasts strains, based on the T_50_ values obtained by Jiranek et al. (1995) (Table 2). Indeed, T_50_ is the time point at which 50% of the initial concentration of the nitrogen source has been assimilated. It can be used to compare assimilation rates. This method can be used to classify YAN sources into different groups according to use preferences (A for the preferential group, B for the intermediate group and C for the non-preferential group). However, the classification of YAN sources within the group may be strain-dependent.

The preferential nitrogen sources identified for *S. cerevisiae* SLM were consistent with published results (Jiranek et al., 1995; Crépin et al., 2012; Kemsawasd et al., 2015). Some nitrogen nutrition data were available for NS yeasts (Andorrà et al., 2010; Kemsawasd et al., 2015). However, Andorrà et al. (2010) did not identify the preferred nitrogen sources of the non-*Saccharomyces* yeasts they studied, and Kemsawasd et al. (2015) used a synthetic must. Our study is the first to provide a global overview of YAN consumption profiles during alcoholic fermentations in grape juice. Kemsawasd et al. (2015) reported that glutamine and ammonium could be considered preferred nitrogen sources for *M. pulcherrima*. Our results suggest that this is indeed the case for glutamine, but not for ammonium. Indeed, we found that ammonium assimilation was affected by temperature. Kemsawasd et al. (2015) identified cysteine, histidine and threonine as “bad” sources of nitrogen for this yeast. We obtained similar results for histidine and threonine at 28°C, but not at 20°C. Furthermore, we obtained the opposite results for cysteine (identified as a preferred source in our study). These comparisons show that the matrix and fermentation conditions affect the regulation of YAN assimilation. These differences may also be due to a strain-dependent effect. The YAN consumption profiles of the three NS yeast strains studied here differed from that of *S. cerevisiae* SLM in terms of both assimilation rates and the types of YAN assimilated. NS yeasts displayed specific patterns of YAN assimilation (non-consumption or preferential nitrogen sources) different from that of *S. cerevisiae*, suggesting that these species may display genomic differences in nitrogen metabolism and regulatory systems. For example, the assimilation, by *P. membranifaciens*, of proline at 28°C, suggests that it may have different mechanisms of transporter regulation, potentially accounting for its effects on the volatile compounds profile of wine (Lambrechts and Pretorius, 2000; Clemente-Jimenez et al., 2005; Sadoudi et al., 2012; Cordero-Bueso et al., 2013; Liu et al., 2016).

For *S. cerevisiae*, the preferential assimilation of certain YAN sources can be explained mainly by the regulation of nitrogen source transport by permeases, including the Ssy1p- Ptr3p-Ssy5 (SPS) system (Ljungdahl, 2009) and the NCR system (Grenson, 1992; Horák, 1997; Crépin et al., 2012; Ljungdahl and Daignan-Fornier, 2012). The recent increase in interest in NS yeasts has not led to explorations of these mechanisms.

Our results also highlight the effect of temperature on YAN assimilation profiles, as already reported for *S. cerevisiae* (Beltran et al., 2007) but never previously reported for non-*Saccharomyces* yeasts. Beltran et al. (2007) suggested that lower temperatures are associated with a lower plasma membrane fluidity and lower levels of molecular motion for phospholipids, membrane proteins and, consequently, some of the permeases involved in YAN transport. These aspects have been described in *S. cerevisiae*, but it is reasonable to assume that the same pattern is likely to occur in NS yeasts.

Sequential inoculation greatly alters nitrogen availability at the time of inoculation with *S. cerevisiae*. The YAN consumed by NS yeasts are not subsequently available to *S. cerevisiae*, and this may affect the concentrations of the volatile compounds generated from these amino acids. For example, leucine, the precursor of the volatile compounds isoamyl acetate in *S. cerevisiae* (Hazelwood et al., 2008) was rapidly consumed by *S. bacillaris* BBMV5FA17 during the first stage of fermentation. *S. bacillaris* has been shown to produce only small amounts of isoamyl acetate (Sadoudi et al., 2012). Thus, in sequential culture, the isoamyl acetate present is produced mostly by *Saccharomyces cerevisiae*. We compared isoamyl acetate production by *S. cerevisiae* in pure and sequential cultures. The levels of this compound were 21 times higher in pure cultures than in sequential cultures, and this difference is probably due to the consumption of its precursor by the NS yeast. Leucine is also the precursor of 2-methylbutanol (Ribéreau-Gayon et al., 2006). We determined the concentration of 2- + 3-methylbutanol, which was significantly lower in sequential fermentations with *S. bacillaris* BBMV5FA17 than in pure cultures of *S. cerevisiae* SLM (Table 3). In this case, as for isoamyl acetate, the decrease in 2- + 3-methylbutanol levels directly reflects the consumption of leucine by *S. bacillaris* BBMV5FA17. Valine was also partially consumed by *S. bacillaris* BBMV5FA17. However, by contrast to our observations for isoamyl acetate and 2- + 3-methylbutanol, the concentration of isobutyl alcohol, the higher alcohol derived from valine (Ribéreau-Gayon et al., 2006), was significantly higher in sequential fermentations (2088.0 ± 225.2 μg/L) than in pure cultures of *S. cerevisiae* SLM (1102.9 ± 292.6 μg/L; Table 3). There are two possible reasons for this: *S. bacillaris* may produce large amounts of isobutyl alcohol or, in cofermentations with *S. cerevisiae*, a synergistic effect results in higher levels of production. Such effects have already been reported (Lambrechts and Pretorius, 2000; Andorrà et al., 2010; Ciani et al., 2010; Sadoudi et al., 2012; Ciani and Comitini, 2015). The same may also be true for sequential fermentations with *M. pulcherrima* BB810, which resulted in significantly higher levels of isobutyl alcohol than were obtained with *S. bacillaris* BBMV5FA17 (Table 3). Similar concentrations of isobutyl alcohol were obtained in pure cultures of *S. cerevisiae* SLM and in sequential fermentations with *P. membranifaciens* BB3 (Table 3). Phenylalanine is another amino acid precursor described in previous studies (Ribéreau-Gayon et al., 2006) and associated with volatile compounds production. All the NS yeasts studied consumed this amino acid in the first part of the sequential fermentation, but, at the end of the sequential fermentation, only *M. pulcherrima* BB810/*S. cerevisiae* SLM sequential fermentations had produced significant amounts of phenylethyl alcohol, the volatile compounds derived from phenylalanine (Table 3). Phenylethyl acetate, an ester derived from phenylethyl alcohol, was present at high concentration (more than twice that in pure cultures of *S. cerevisiae* SLM). *S. cerevisiae* is known to produce larger amounts of phenylethyl acetate than pure cultures of NS yeasts, but even higher phenylethyl acetate levels were reported in a previous study for a *M. pulcherrima*/*S. cerevisiae* coculture (Sadoudi et al., 2012). In this study, other than for leucine and valine, the link between amino acid concentration and volatile compound levels is not straightforward. Indeed, the production of higher alcohols and esters by yeasts is a highly complex process dependent on a number of parameters. Higher alcohols are produced either from their amino acid precursors or by central carbon metabolism (Lambrechts and Pretorius, 2000; Swiegers and Pretorius, 2005; Mouret et al., 2009; Styger et al., 2011) showed that isobutanol and isoamyl alcohol were not produced exclusively from their amino acid precursors. The availability of lipids and of oxygen also affects acetate formation. The *de novo* synthesis of acetate by yeast is restricted if lipids are abundant in the growth medium. This, in turn, reduces the production by the yeast of related aroma compounds, such as fatty acid ethyl esters and isoamyl acetate (Yunoki et al., 2005; Rollero et al., 2015). In addition to the specific metabolic features of individual strains, complex interactions between yeasts involving competition for nitrogen, lipids, and oxygen, also control the concentrations of esters and higher alcohols in sequential culture. Finally, the production of volatile compounds by yeasts is dependent on the types and amounts of YAN in the medium (Bell and Henschke, 2005; Torrea et al., 2011). As demonstrated here, non-*Saccharomyces* yeasts have quantitative and qualitative effects on YAN, at least partly explaining the differences in compound concentrations between pure cultures of *Saccharomyces* and sequential fermentations.

In conclusion, we provide here the first characterization of the specific nitrogen preferences of NS yeast strains. Our results highlight considerable differences in preferred nitrogen sources between the species studied. Further studies are required to determine the molecular basis of these differences. Our findings highlight the need to monitor nitrogen levels in sequential fermentations for winemaking, to prevent sluggish *S. cerevisiae* fermentations due to the depletion of YAN by non-*Saccharomyces* yeasts. From these results, a specific addition of amino acids in must could be considered. Moreover the impact of the sequential fermentation on the volatile and aromatic profile deserves further investigation. In a context of indigenous fermentation, our work suggests that non-*Saccharomyces* yeasts compete with *Saccharomyces* for nitrogen sources, with implications for the organoleptic characteristics of the wines produced.

## Author contributions

Conceived and designed the study: AG, HA, RT, CM, and CS. Performed nitrogen HPLC experiments: AG, YL. Developed analysis method for amino acids and ammonium integration: AG, YL, HA, and RT. Determination of the concentration of volatile constituents in the wine and analysis: SV, BQ, AG, HA, RT. Drafted the manuscript: AG. Corrected and refined the manuscript: HA, RT, CM, CS, YL, SV, and BQ. All authors read and approved the final manuscript.

### Conflict of interest statement

The authors declare that the research was conducted in the absence of any commercial or financial relationships that could be construed as a potential conflict of interest.
