# Supplementary material for: Non-Saccharomyces Yeasts Nitrogen Source Preferences: Impact on Sequential Fermentation and Wine Volatile Compounds Profile
Source: Front Microbiol. 2017 Nov 6;8:2175. doi: 10.3389/fmicb.2017.02175 (PMC5672154; doi:10.3389/fmicb.2017.02175)
Supplement: Supplementary file 1 [file Presentation1.PDF]

Supplementary Material

Non-*Saccharomyces* yeasts nitrogen source preferences: impact on sequential fermentation and wine volatile compounds profile

Antoine Gobert[1*], Raphaëlle Tourdot-Maréchal[1], Christophe Morge[2], Céline Sparrow[2] Youzhong Liu[1], Beatriz Quintanilla-Casas[2], Stefania Vichi[2] and Hervé Alexandre[1]

*** Correspondence:** antoinegobert1@gmail.com

1 Supplementary Figures

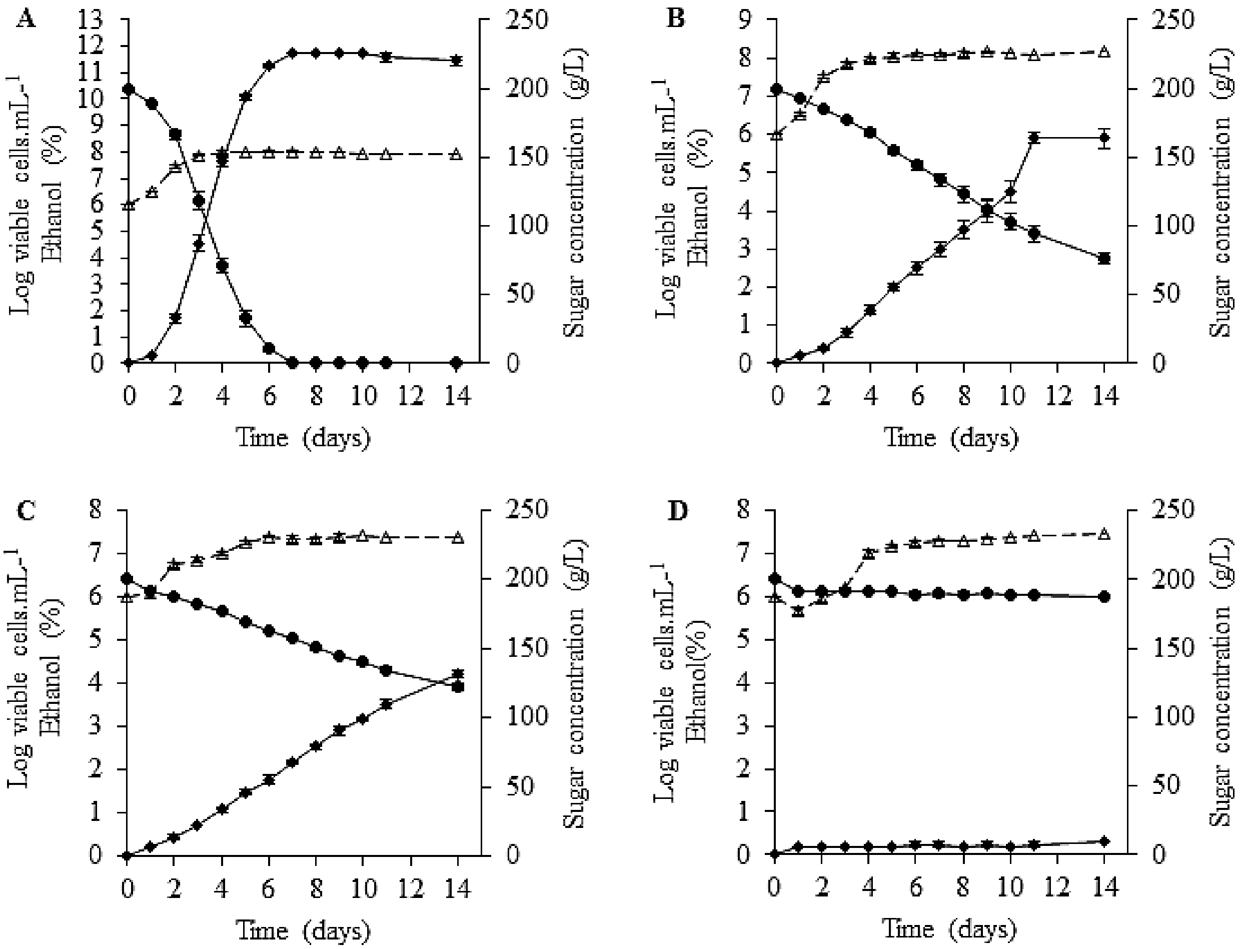

Supplementary figure 1: Fermentation profiles of selected NS yeasts in grape juice in static condition at 20°C. **(A)** *S. cerevisiae* SLM, **(B)** *S. bacillaris* BBMV5FA17, **(C)** *M. pulcherrima* BB810 and **(D)** *P. membranifaciens* BB3. Dotted curves with white triangles represent viable populations, solid curves with black circles show sugar concentration and solid curves with black diamonds indicate the concentration of ethanol. For each strain, the experiments were performed in triplicate and the error bars represent the standard deviation of the results.

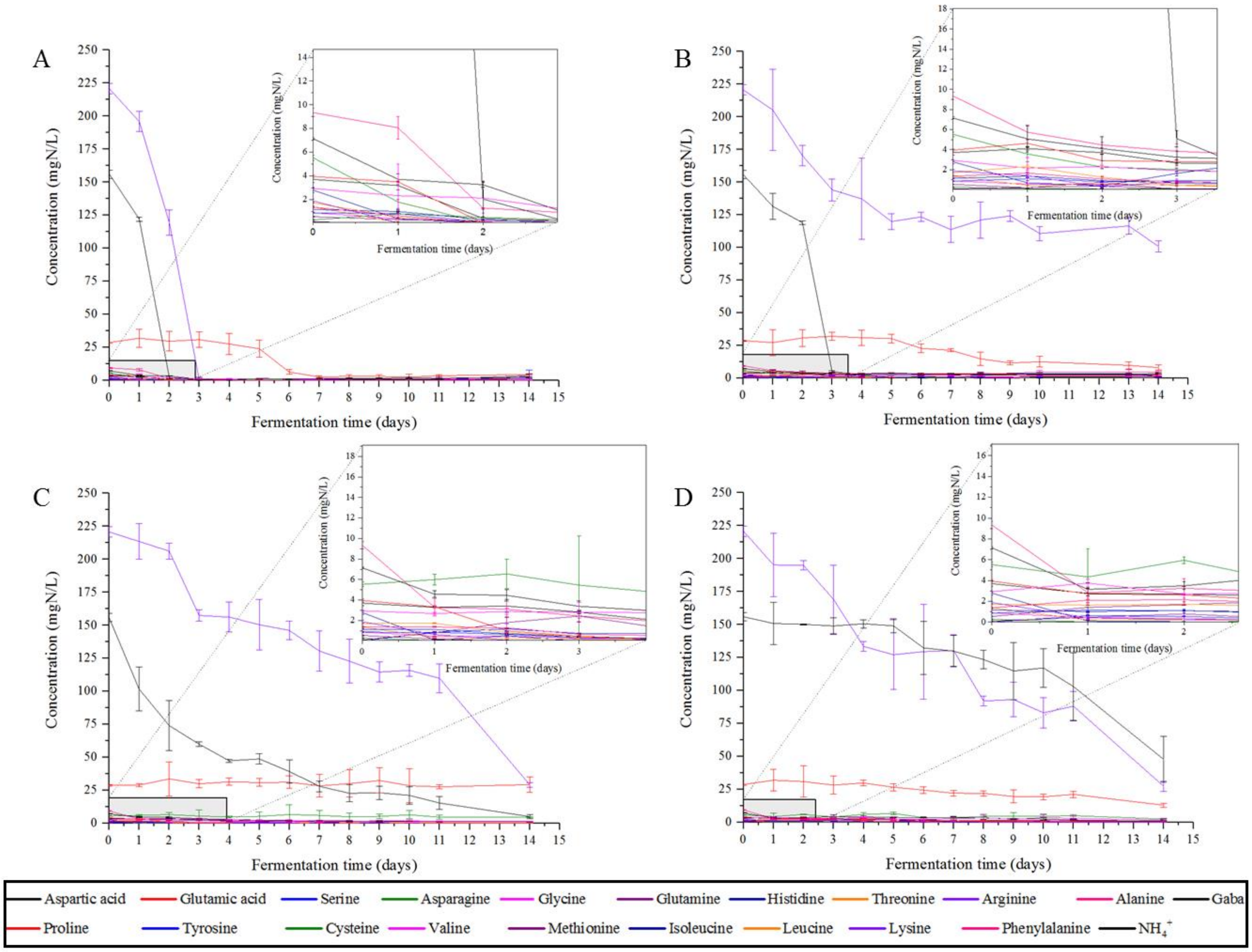

Supplementary figure 2: YAN consumption profiles of *S. cerevisiae* SLM **(A)**, *S. bacillaris* BBMV5FA17 **(B)**, *M. pulcherrima* BB810 **(C)** and *P. membranifaciens* BB3 **(D)** in grape juice at

20°C. For each strain, the experiments were performed in triplicate and the error bars represent the standard deviation of the results.

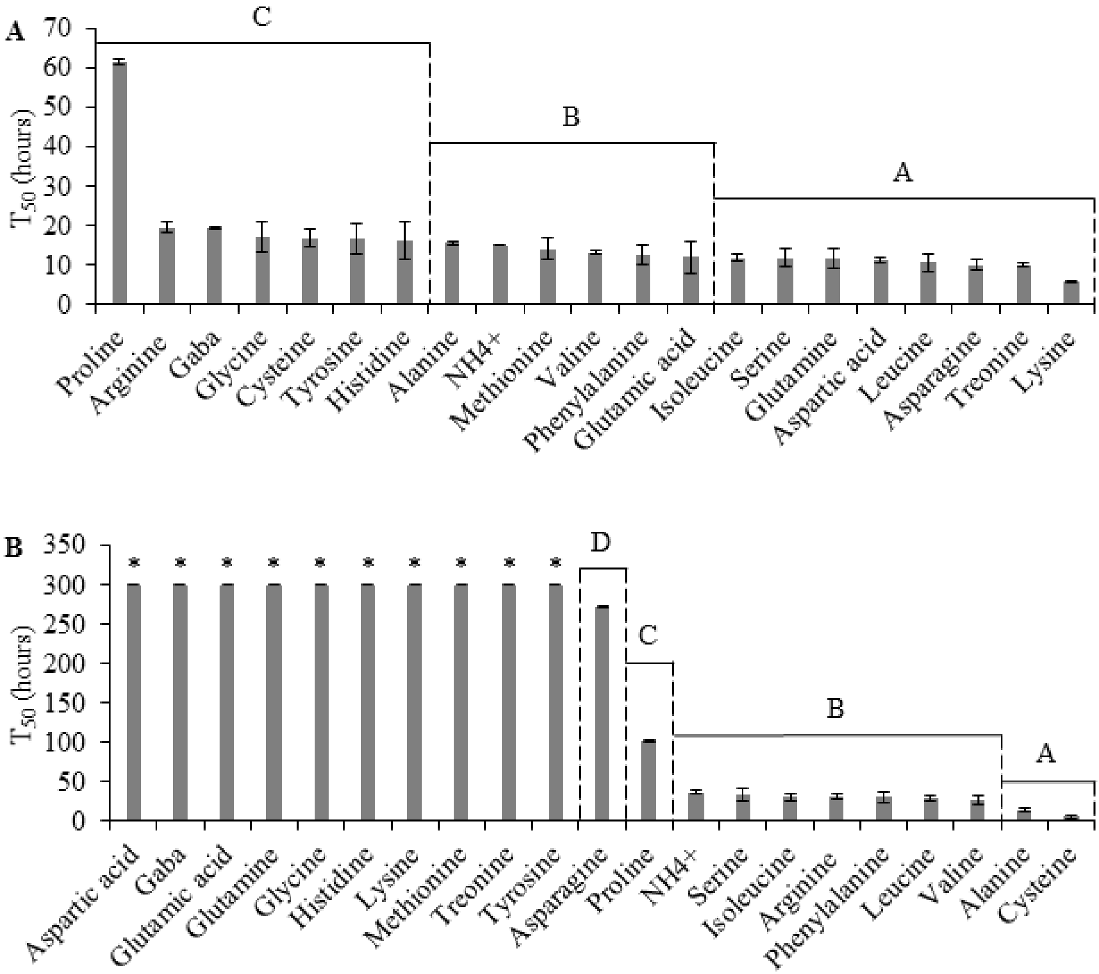

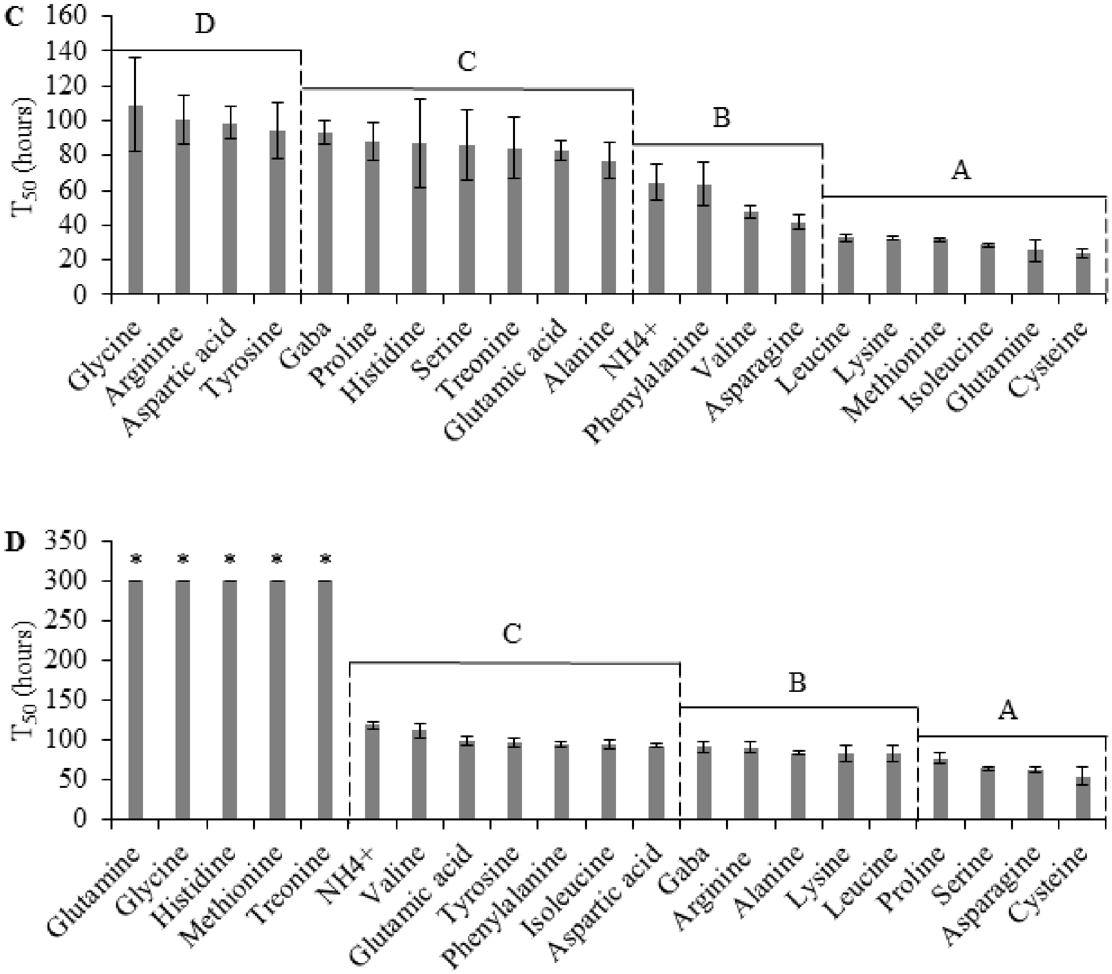

Supplementary figure 3: YAN classification according their T_{50} value for **(A)** *S. cerevisiae* SLM, **(B)** *S. bacillaris* BBMV5FA17, **(C)** *M. pulcherrima* BB810 and **(D)** *P. membranifaciens* BB3 in grape juice at 28°C. In each panel, the group A represents preferential source, B intermediate source, C non-preferential source and D or * a very low assimilation or not assimilation. All fermentations were performed in duplicate and error bars represent standard deviations of the results. Groups of YAN sources represented by letters were significantly different (Kruskal-Wallis test, $p < 0.05$).

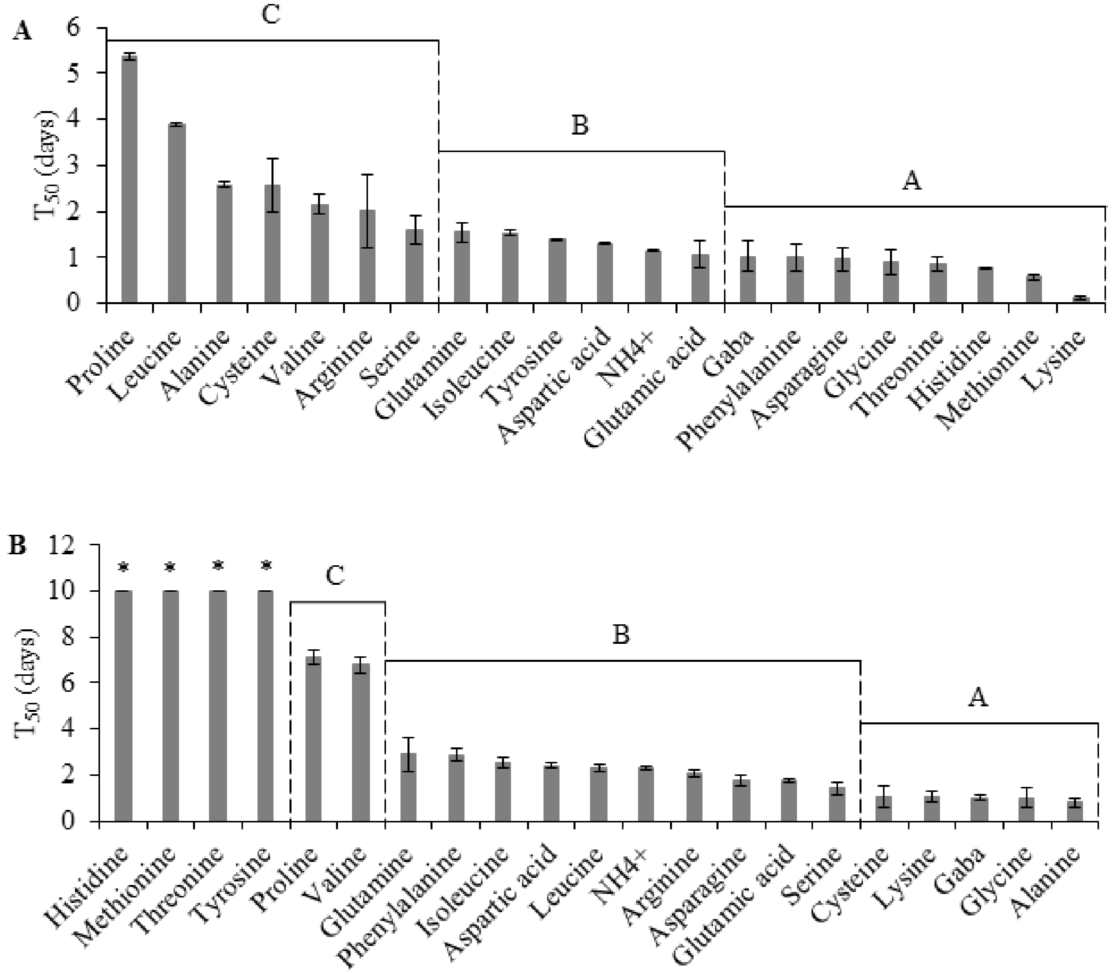
A
B
T50 (days)
C
B
A
Proline
Leucine
Alanine
Cysteine
Valine
Arginine
Serine
Glutamine
Isoleucine
Tyrosine
Aspartic acid
NH4+
Glutamic acid
Gaba
Phenylalanine
Asparagine
Glycine
Threonine
Histidine
Methionine
Lysine
Histidine
Methionine
Threonine
Tyrosine
Proline
Valine
Glutamine
Phenylalanine
Isoleucine
Aspartic acid
Leucine
NH4+
Arginine
Asparagine
Glutamic acid
Serine
Cysteine
Lysine
Gaba
Glycine
Alanine

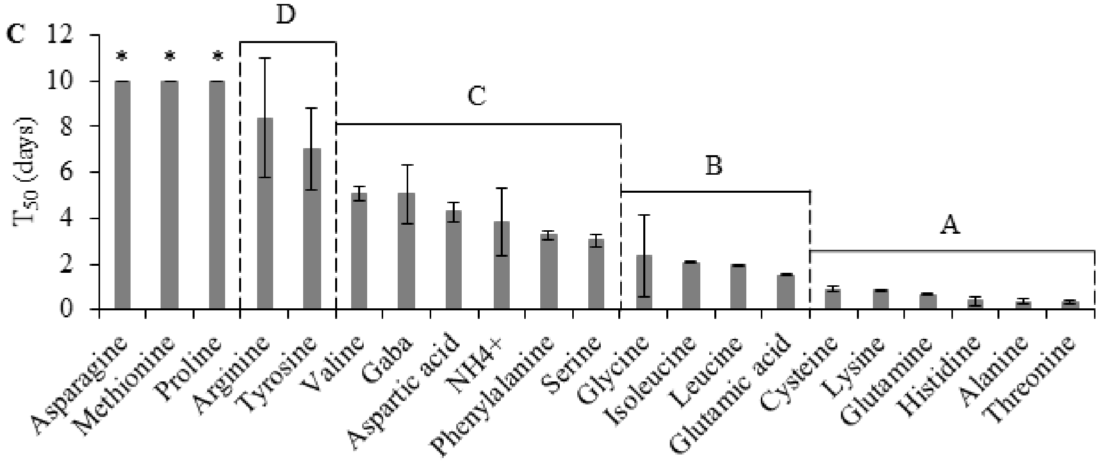

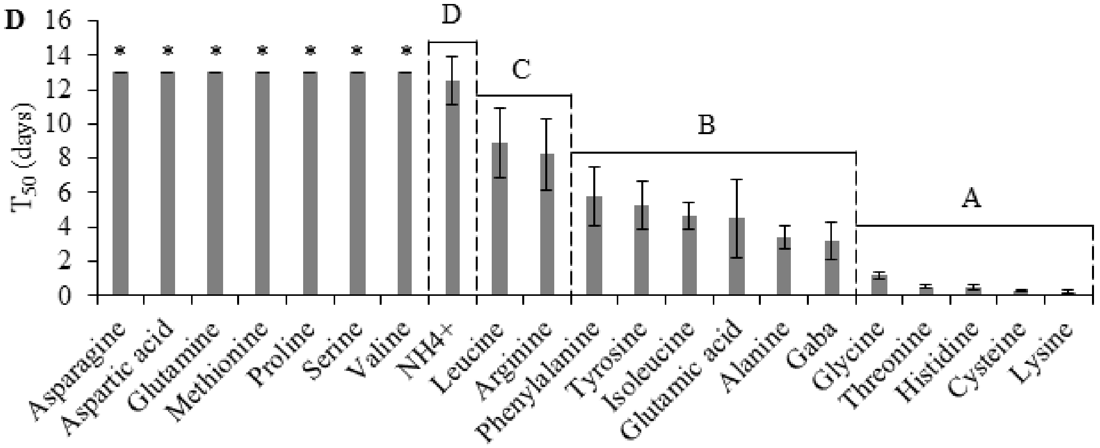

Supplementary figure 4: YAN classification according their T_{50} value for **(A)** *S. cerevisiae* CEPPO 8260, **(B)** *S. bacillaris* BBMV5FA17, **(C)** *M. pulcherrima* BB810 and **(D)** *P. membranifaciens* BB3 in grape juice at 20°C. In each panel, the group A represents preferential source, B intermediate source, C non-preferential source and D or * a very low assimilation or not assimilation. All fermentations were performed in triplicate and error bars represent standard deviations of the results. Groups of YAN sources represented by letters were significantly different (Kruskal-Wallis test, $p < 0.05$).
